# GENETIC EPIDEMIOLOGY OF ANKLE BRACHIAL INDEX IN THE LONG LIFE FAMILY STUDY

**DOI:** 10.1093/geroni/igad104.1970

**Published:** 2023-12-21

**Authors:** Deidra Ressler, Ryan Cvejkus, Warwick Daw, Mary Feitosa, Joanne Murabito, Ryan Minster, Joseph Zmuda, Allison Kuipers

**Affiliations:** University of Pittsburgh School of Public Health, Pittsburgh, Pennsylvania, United States; University of Pittsburgh School of Public Health, Pittsburgh, Pennsylvania, United States; Washington University School of Medicine, St. Louis, Missouri, United States; Washington University School of Medicine, St. Louis, Missouri, United States; Boston University Chobanian & Avedisian School of Medicine, Boston, Massachusetts, United States; University of Pittsburgh School of Public Health, Pittsburgh, Pennsylvania, United States; University of Pittsburgh School of Public Health, Pittsburgh, Pennsylvania, United States; University of Pittsburgh School of Public Health, Pittsburgh, Pennsylvania, United States

## Abstract

The genetic influence on peripheral vascular health in exceptional aging has not been well-studied. Thus, we performed heritability, genome-wide linkage analyses (GWLA) and genome-wide association studies (GWAS) of ankle-brachial index (ABI) and peripheral artery disease (PAD) in 3006 individuals from the Long Life Family Study (LLFS). The LLFS is a longitudinal, family-based study including long-lived siblings and their families. ABI was measured in-home, and the minimum from both sides was used, with exclusions for ABI≥1.4 or non-compressible arteries. PAD was defined as ABI<0.9. We performed heritability and GWLA using SOLAR and GWAS using R accounting for familial relationships. Our full model was adjusted for age, sex, site, significant PCs, blood pressure, and relevant risk factors including smoking, body size, and lipids. We considered significance at a LOD≥3.0 for GWLA and at a p<5x10-8 for GWAS. The proband generation was 53.2% female, aged 88.7 and had a mean ABI of 1.06. The offspring generation was 60.1% female, aged 60.1 and had a mean ABI of 1.19. 18.2% of probands and 1.0% of offspring had PAD (7.4% overall). Residual genetic heritability was 0.12 (p=8.7x10-4) for ABI and 0.23 for PAD (p=0.0662). We found linkage with chromosome 15q12 (LOD=3.65) for ABI and association on chromosome 4q25 (rs12512857; p=6.25x10-8; ANK2; maf=0.021) for PAD. ANK2 is a gene that has been implicated in cardiac dysfunction though there have been no previous associations with ABI or PAD. This suggests that unique genetic variation is associated with peripheral vascular health in long-lived families.

